# A 20-year dataset (2001–2020) of global cropland water-use efficiency at 1-km grid resolution

**DOI:** 10.1038/s41597-025-04904-1

**Published:** 2025-04-04

**Authors:** Min Jiang, Chaolei Zheng, Li Jia, Jiu Chen

**Affiliations:** 1 International Research Centre of Big Data for Sustainable Development Goals, Beijing, 100094 China; 2 State Key Laboratory of Remote Sensing and Digital Earth, Aerospace Information Research Institute, Chinese Academy of Sciences, Beijing, 100101 China

**Keywords:** Hydrology, Hydrology

## Abstract

Cropland water-use efficiency (WUE) is an essential indicator for the sustainable utilization of agricultural water resources. The lack of long-term global cropland WUE datasets with high spatial resolution limits our understanding of global and regional patterns of cropland WUE. This study developed a long-term global cropland WUE dataset at 1-km spatial resolution from 2001 to 2020. The cropland WUE was obtained as the ratio between net primary productivity (NPP) and evapotranspiration that was retrieved from ETMonitor global evapotranspiration datasets. The global cropland NPP was estimated by subtracting plant respiration from gross primary production (GPP), which was estimated using an improved light-use efficiency model after being optimized for different global climate zones using flux-tower observation data. The generated WUE product showed good accuracy with high correlation efficiency (0.76) and low root mean square error (0.5 g C/kg H_2_O/yr) compared with the ground measurements at flux towers. This dataset can be used as fundamental data to advance the efficient utilization of water use for sustainable development.

## Background & Summary

To address the urgent issue of water scarcity at regional and global scales, “Change in water-use efficiency over time” is listed as one of the important indicators of the United Nations Sustainable Development Goal SDG 6.4^1^. Agriculture is a major consumer of freshwater and accounts for more than 90% of the global consumptive water footprint^2^. By reducing the amount of water used per unit of productivity, improving the water-use efficiency (WUE) of cropland is one of the most important ways to cope with water scarcity^3^.

WUE refers to the biomass yield or economic value produced per unit of water, which can reflect the biological and economic benefits, taking into account both input and output aspects^4^. Cropland WUE can be expressed in different ways, such as the ratio of cropland gross primary productivity (GPP) and water consumption through evapotranspiration (ET)^5–7^, the ratio of cropland net primary productivity (NPP) and ET^8,9^, and the ratio of cropland yield and ET^3,10^, etc. Cropland WUE can comprehensively reflect the trade-off relationship between food production from cultivated land and water consumption, and it is an effective index to guide the sustainable utilization of regional agricultural water resources^11,12^.

Previous studies have evaluated cropland WUE based on plot-scale observations or social statistical data, which were insufficient in terms of spatial-temporal coverage, timeliness, update frequency, etc., and these limit their capability to reflect the spatial-temporal variation characteristics of cropland WUE in large regions^13^. Satellite remote sensing technology can be used to monitor regional cropland WUE over a large area for a long period^4,14^, because it can provide timely and large-scale land surface variables that server as inputs for WUE estimation. Various methods have been developed to estimate WUE using remote sensing data^14^. Several empirical models are developed using simple linear regression based on meteorological variables and remote sensing vegetation indices (VIs) have been developed and applied in many regions^15,16^. However, the relationship between WUE and VIs differed significantly across biomasses and regions^17^, which limits the global applications of these empirical models and may lead to the failure to capture the complex relationship between WUE and VIs^14^. Analytical models also provided a promising solution to estimate WUE and improve our understanding of ecosystem carbon-water cycle coupling^18^, while the assumptions of these models need to be tested and validated^14,18^. With the boom in remote sensing technology, significant improvements have been made in the algorithms and continuous datasets of vegetation production (GPP, NPP, etc.) and water consumption (e.g., ET), which conceptually provide new opportunities for estimating WUE. And recent studies have increasingly estimated the global ecosystem WUE as the ratio of GPP (or NPP) to ET, which were independently obtained based on satellite remote sensing^13,19^. Although the superiority of remote sensing technology in the assessment of cropland WUE has been recognized^14,20^, its widespread application and reliability are limited, and there is a lack of a long-term spatially and temporally continuous dataset of global and regional cropland WUE.

The reliability of the estimated WUE is highly dependent on the accuracy of the crop production process simulations, which is a major challenge for global WUE estimation. Vegetation productivity and ET are two of the most important basic variables for WUE estimate. Vegetation productivity (GPP or NPP) is commonly estimated based on the Light-use-efficiency model (LUE model)^21^, and many regional and global vegetation productivity datasets have been generated accordingly, such as the Moderate Resolution Imaging Spectroradiometer (MODIS) GPP/NPP algorithm and products (i.e., MOD17). Previous studies have shown that MOD17 severely underestimates the total GPP in croplands^22^ and fails to capture the seasonal and inter-annual changes, mainly due to the inadequate simulation of the effect of water availability on GPP^23^. In our previous study, an improved EF-LUE model, which uses the evaporative fraction (EF) to reflect the regulation of plant water availability, was developed and found to be more reliable for simulating cropland GPP because it can account for the coupling effect between the carbon assimilation and water use^24^. There are also many remote sensing-based global ET products, among which the MOD16 ET^25,26^ and the Global Land Evaporation Amsterdam Model (GLEAM) ET^27^ are the most well-known, which still have many limitations when applied for WUE estimation, such as the spatiotemporal resolution and accuracy. For example, the GLEAM ET has a coarse spatial resolution (0.25°), which is not suitable for the generation of moderate-resolution WUE datasets for cropland^10^. The MOD16 ET product has a finer spatial (500 m) and temporal (8-day) resolution, but previous studies have shown that the MOD16 ET product has a relatively large bias in the arid and semi-arid regions and a significant underestimate of irrigated croplands ET due to its inadequate consideration of soil moisture constraints on ET^10,28^, which may lead to an overestimate of cropland WUE. The recently developed ETMonitor global ET with daily 1-km resolution is spatially and temporally continuous with good accuracy^29^. In particular, this dataset excels in representing the spatial variation of ET in the irrigated cropland regions compared to other global ET products (such as GLEAM ET and Penman-Monteith-Leuning (PML-V2) ET product), which are helpful for estimating WUE. In addition, previous studies also suggest that there is an internal inconsistency between the estimation of ET and GPP, which is caused by the difference between the forcing for GPP and ET when they are estimated independently^30^.

Another major challenge comes from the differences in plant characteristics between different climate zones^31^, which have rarely been taken into account in remote sensing-based estimation of global WUE, GPP, and ET^32^. For example, previous studies show the large spatial variability in the potential LUE and stomatal conductance within individual biome types in different climate zones^33^. Fortunately, this has been recognized in the ETMonitor ET, which calibrated the most sensitive parameters for different land cover types and climate regions according to in-situ flux tower observations^29^. The parameters of the EF-LUE model have also been optimized using the flux-tower observations, and the optimized parameters are spatially extrapolated according to climate zones for global scale application^31^. The integration of ETMonitor ET and the optimized EF-LUE model has a promising performance in simulating spatial and temporal variations of global cropland GPP and ET, and thus can contribute to improve the global cropland WUE estimation.

In this study, we developed a 20-year (2001–2020) global cropland WUE dataset with a spatial resolution of 1-km by combining the improved EF-LUE model and the ETMonitor model, driven by consistent forcing data and considering the coupling relationship between the carbon and water exchange. This dataset can help to evaluate and explore the changes in the global cropland WUE and support the monitoring and assessment of the SDG 6.4.

## Methods

### Estimation of cropland water-use efficiency

WUE can be represented by various ratios, such as GPP/ET and NPP/ET. For assessing cropland water use efficiency, the NPP/ET ratio is more appropriate, as it directly indicates the net carbon sequestrated in harvestable yield. By contrast, GPP involves respiratory losses associated with biomass growth, especially under abiotic stressors like extreme temperatures or soil moisture deficits, which can disproportionately increase respiration and weaken the link between GPP and actual productivity. Therefore, in this study, we defined cropland WUE as the ratio of NPP to ET:

1 $${WUE}=\frac{{NPP}}{{ET}}$$

where NPP is the annual cumulative net primary productivity (g C/m^2^/yr), and ET is the annual cumulative evapotranspiration (mm/yr) of cropland.

**Fig. 1:**
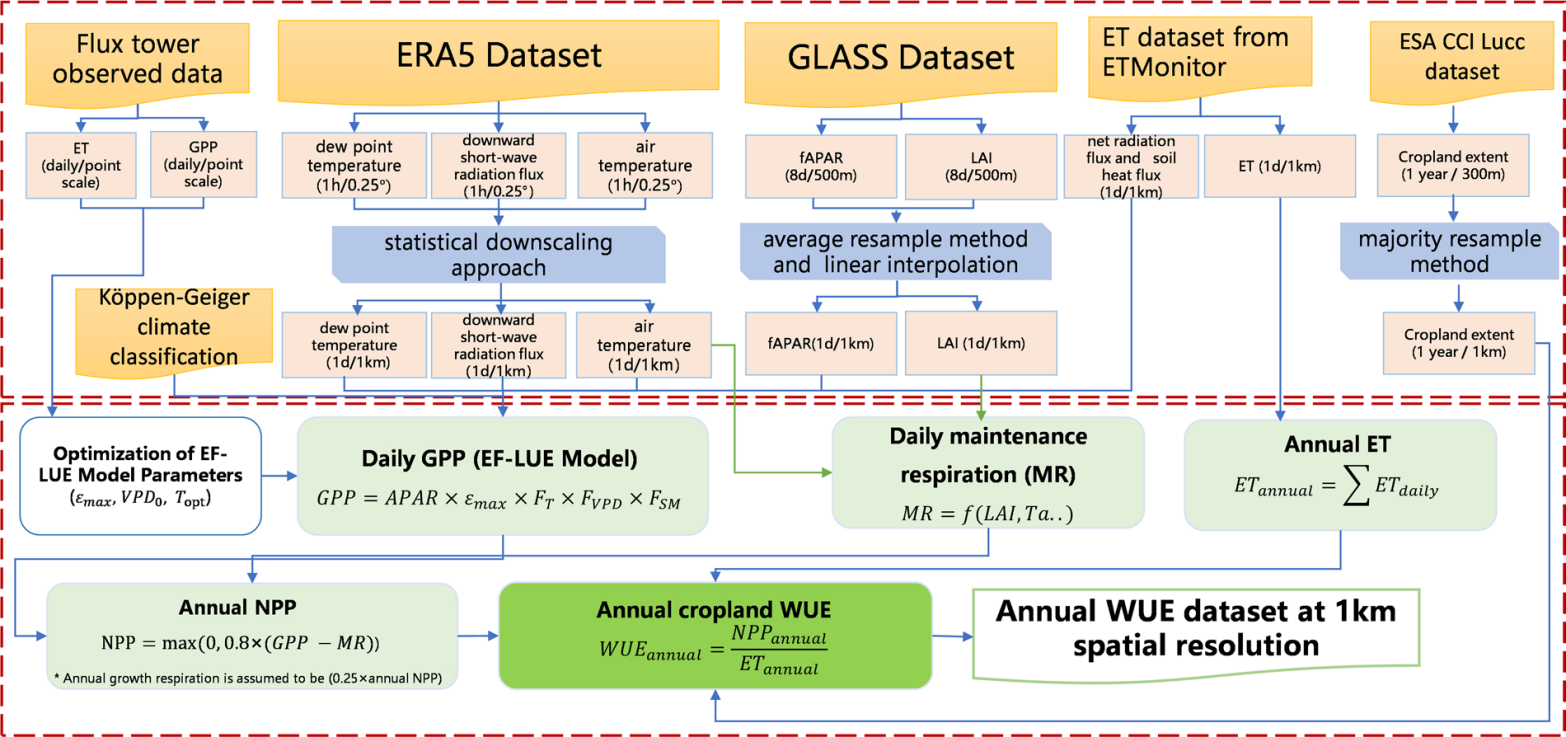
Schematic overview of the study workflow for global cropland WUE estimate.

In this study, we used ET from ETMonitor to calculate the water use of cropland and utilized an improved EF-LUE model to estimate GPP and then NPP. In addition, the ET from the ETMonitor model was used to form a water stress factor to improve the GPP estimate in the EF-LUE model, reflecting the coupled relationship between carbon exchange and water exchange. This practice of using the same forcing datasets for GPP estimation with the common input variables of ETMonitor and the improved EF-LUE model by introducing EF as water stress factor in GPP estimation also can also avoid the uncertainties in WUE estimation caused by different forcing. The estimation of WUE for cropland consisted of three main steps (Fig. 1):

- Cropland ET was obtained from the global daily ET dataset with 1 km spatial resolution produced by the ETMonitor model by using multiple remote sensing data and validated using ground flux measurements at flux tower sites. The daily ET data were aggregated to an annual sum.
- The NPP is equal to GPP minus Crop Respiration (CR). GPP was estimated using an improved Light Use Efficiency model (EF-LUE)^31^, which introduced a new parameterization scheme to account for the soil water stress, thus improving the accuracy of GPP estimation under different soil water conditions. CR was obtained by summing the maintenance respiration (related to Leaf Area Index, etc.) and growth respiration (proportional to NPP) using an analogous algorithm and parameter scheme of the MOD17A2H/A3H product.
- The annual NPP and annual ET were used to calculate the annual WUE of cropland. The annual WUE was then validated by using the flux tower observation data.

### Estimation of gross primary productivity

In this study, the improved light-use efficiency model developed in our previous study^31^, called the EF-LUE model, was used to estimate GPP. The EF-LUE model showed better performance mainly because of the following two aspects: (1) soil water stress factors were introduced in the EF-LUE model to improve the accuracy of estimated GPP under conditions with non-optimal water supply, and (2) model parameters were calibrated and optimized for different climate zones based on observations of eddy covariance system from global flux towers, and the optimized parameters were interpolated for global GPP estimation. In the EF-LUE model, GPP is the product of the actual LUE and APAR. Actual LUE is the maximum LUE constrained by environmental conditions including air temperature, VPD, and water availability. GPP (g C/m^2^/d) is calculated as:

2 $${GPP}={fAPAR}\times {PAR}\times {\varepsilon }_{\max }\times {F}_{T}\times {F}_{{VPD}}\times {F}_{W}$$

where *f*APAR is the fraction of absorbed photosynthetically active radiation. PAR is the photosynthetically active radiation (MJ/m^2^/d) estimated as a fraction (set to 0.48 in this study) of total shortwave solar irradiance. ${\varepsilon }_{\max }$ is the maximum LUE in the absence of environmental stresses (g C/MJ), and the values were optimized using ground site data and interpolated according to climate zones for GPP estimation at a global scale.

*F*_*T*_ is the air temperature constraint factor and is calculated as:

3 $${F}_{T}=\frac{\left(T-{T}_{\min }\right)\times \left(T-{T}_{\max }\right)}{\left(T-{T}_{\min }\right)\times \left(T-{T}_{\max }\right)-{(T-{T}_{{opt}})}^{2}}$$

where *T* is the daily average air temperature (°C); ${T}_{\min }$, ${T}_{\max }$, and ${T}_{{opt}}$ are the minimum, maximum, and optimum air temperatures (°C) for vegetation photosynthesis, respectively. ${F}_{T}$ were set to 0 when the average temperature *T* is lower than ${T}_{\min }$ or higher than ${T}_{\max }$, resulting in “0” LUE. In this study, ${T}_{\min }$ and ${T}_{\max }$ were set to 0 °C and 40 °C, respectively; ${T}_{{opt}}$ is optimized using CO_2_ flux measurements collected at flux tower sites characterized as cropland.

*F*_*VPD*_ is the atmospheric water Vapor Pressure Deficit (VPD) constraint factor and is calculated as:

4 $${F}_{{VPD}}=\frac{{{VPD}}_{0}}{{{VPD}}_{0}+{VPD}}$$

5 $${VPD}=0.6108\times \left({e}^{\frac{12.27\times T}{T+237.3}}-{e}^{\frac{12.27\times {Td}}{{Td}+237.3}}\right)$$

where *VPD*_0_ is the half-saturation coefficient (kPa) and is obtained by the optimization procedure described in Section *“Parameters optimization of EF-LUE model and global application”* of this study. VPD is the atmospheric water Vapor Pressure Deficit (kPa). *T* and *Td* are the daily average air temperature (°C) and the dew-point temperature (°C), respectively.

*F*_*W*_ is the water availability constraint factor, and is calculated as:

6 $${F}_{W}=\min \left\{1,\max \left(0,{EF}\right)\right\}$$

7 $${EF}=\frac{\lambda {ET}}{{R}_{n}-G}$$

where *λET* is the latent heat flux (W m^−2^) associated with ET. *R*_*n*_ is the net radiation flux (W m^−2^), and *G* is the soil heat flux (W m^−2^). As the EF-LUE model works at a daily time scale, the *R*_*n*_ and *G* are taken as mean daily values.

The GPP was estimated at a daily step, and then the daily GPP in a calendar year was aggregated to annual GPP to calculate the annual cropland WUE.

### Parameters optimization of EF-LUE model and global application

In this study, we applied an optimization procedure to estimate the aforementioned parameters in the EF-LUE model^31^, including ${\varepsilon }_{\max }$, ${T}_{{opt}}$, and ${{VPD}}_{0}$, using site-level data. The optimization was performed for each site in different climate zones using the Trust Region Reflective algorithm which is a nonlinear fitting algorithm to robustly solve bounded problems^34^. It obtains the optimized parameter value by minimizing the sum of squared residuals between the model estimation and the observation (GPP in this study). At each site, all available data were used to optimize the model parameters, to obtain the best parameters. The initial value and the lower and upper bounds used to optimize the three model parameters are shown in Table 1.

**Table 1:** Initial values and lower and upper bounds of each parameter used for EF-LUE model optimization.

parameter	Initial value	Upper bound	Lower bound
*ε*_*max*_ (g C/MJ)	3.82	4	0
*T*_*opt*_ (°C)	28.0	35	0
*VPD*_0_ (kPa)	1.2	3	0

**Fig. 2:**
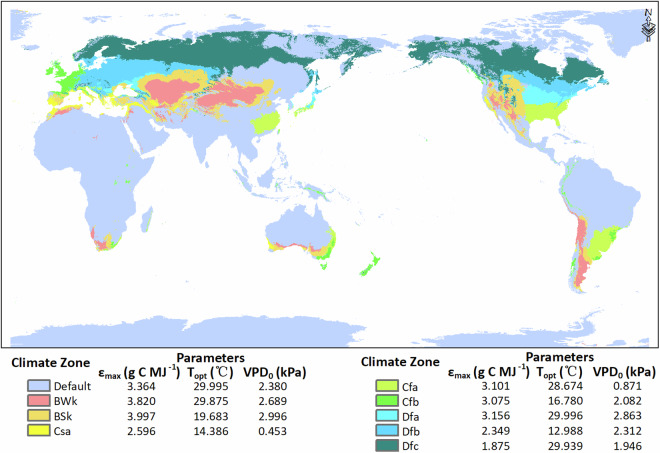
Optimized model parameters of the EF-LUE model for cropland GPP estimate in different climate zones on a map of Köppen climate zones used in the current study. Codes of climate zones in the color legend are adopted from Köppen-Geiger climate classification. The “Default” region means the climate zones without cropland sites of flux tower observation.

In the global pixel-wise GPP calculation, the optimized parameters are spatially interpolated according to the climate classification: for a climate zone with flux tower measurements, the model parameters were optimized using ground measurements from all available sites in the corresponding climate zone; for a climate zone without flux tower measurement data, the model parameters were optimized by using the ground measurements from all available cropland sites worldwide (called “Default” values of the parameters). The available cropland sites are mainly located in dry, temperate, and continental climate zones based on Köppen-Geiger climate classification^35^. The optimized parameters of each climate zone are shown in Table 2 and Fig. 2.

**Table 2:** Optimized model parameters of the EF-LUE model for cropland GPP estimate in different climate zones (based on Köppen-Geiger climate classification).

Climate Zone	*ε*_*max*_ (g C MJ^−1^)	*T*_*opt*_ (°C)	*VPD*_0_ (kPa)
Bwk	3.820	29.875	2.689
BSk	3.997	19.683	2.996
Csa	2.596	14.386	0.453
Cfa	3.101	28.674	0.871
Cfb	3.075	16.780	2.082
Dfa	3.156	29.996	2.863
Dfb	2.349	12.988	2.312
Dfc	1.875	29.939	1.946
Default	3.364	29.995	2.380

### Respiration estimation

Crop respiration includes maintenance respiration (MR) and growth respiration (GR). In this study, we used an algorithm and parameter scheme similar to the MOD17 NPP product^36^ to estimate crop respiration.

MR is the energy cost of the crop to support life. MR is very sensitive to temperature changes, and different organs have different temperature sensitivity. In this study, we calculated the daily MR of fine roots and leaves using the following equations:

8 $${MR}={Lea}{f}_{{MR}}+{{Froot}}_{{MR}}$$

9 $${Lea}{f}_{{MR}}={Lea}{f}_{{Mass}}\times {Leaf}{\rm{\_}}{mr}{\rm{\_}}{base}\times {Q}_{10{\rm{\_}}{leaf}{\rm{\_}}{mr}}^{\left[\frac{{Ta}-20}{10}\right]}$$

10 $${Q}_{10{\rm{\_}}{leaf}{\rm{\_}}{mr}}=3.22-0.046\ast {Ta}$$

11 $${Lea}{f}_{{Mass}}={LAI}/{SLA}$$

12 $${{Froot}}_{{MR}}={{Fine}{\rm{\_}}{Root}}_{{Mass}}\times {Froot}{\rm{\_}}{mr}{\rm{\_}}{base}\times {Q}_{10{\rm{\_}}{froot}{\rm{\_}}{mr}}^{\left[\frac{{Ta}-20}{10}\right]}$$

13 $${{Fine}{\rm{\_}}{Root}}_{{Mass}}={Lea}{f}_{{Mass}}\times {Froot}{\rm{\_}}{leaf}{\rm{\_}}{ratio}$$

where *Leaf*_*MR*_ and *Froot*_*MR*_ is the MR of leaves and fine roots respectively (kg C kg/C/d). *Leaf*_*Mass*_ is the leaf mass (kg). *Leaf_mr_base* is the MR per unit leaf carbon per day at 20 °C and was set to 0.0098 in this study. *Q*_*10_leaf_mr*_ is the temperature sensitivity of leaf respiration and is a function of the daily average temperature. Ta is the daily average temperature. LAI is the leaf area index (m^2^ leaf m^−2^ ground area). SLA is the specific leaf area (projected leaf area per unit mass of leaf C, m^2^ kg/C) and was set to 30.4 in this study. Fine_Root_Mass_ is the fine root mass (kg). *Froot_mr_base* is the MR per unit fine root carbon per day at 20 °C and was set to 0.00819 in this study. *Froot_leaf_ratio* is the ratio of fine root carbon to leaf carbon and was set to 2.0 in this study. *Q*_*10_froot_mr*_ is the temperature sensitivity of fine root respiration and it was set to 2.0, a constant, in this study. Daily MR in a calendar year was then aggregated to annual MR.

GR refers to the energy consumed by crops to produce organic compounds. Annual growth respiration was empirically parameterized as 25% of annual NPP^37,38^:

14 $${GR}=0.25\times {NPP}$$

where NPP is annual net primary productivity (g C/m^2^/d), and GR is annual crop growth respiration (g C/m^2^/d).

### Net primary productivity estimation

Annual NPP was estimated by the difference between annual GPP and respiration (including MR and GR):

15 $${NPP}=\mathop{\sum }\limits_{i=1}^{n}{{GPP}}_{i}-\mathop{\sum }\limits_{i=1}^{n}{{MR}}_{i}-{GR}$$

where *i* represents the *i*-day in the year with total *n* days (n = 365 for normal year and 366 for leap year).

Combining Eq. (14) and Eq. (15), annual NPP (g C/m^2^/d) is estimated as:

16 $${NPP}=\mathop{\sum }\limits_{i=1}^{n}{{GPP}}_{i}-\mathop{\sum }\limits_{i=1}^{n}{{MR}}_{i}-0.25\times {NPP}=0.8\times \left(\mathop{\sum }\limits_{i=1}^{n}{{GPP}}_{i}-\mathop{\sum }\limits_{i=1}^{n}{{MR}}_{i}\right)$$

The NPP value cannot be less than 0, so annual NPP (g C/m^2^/d) is finally estimated as:

17 $${NPP}=\max \left(0,0.8\times \left(\mathop{\sum }\limits_{i=1}^{n}{{GPP}}_{i}-\mathop{\sum }\limits_{i=1}^{n}{{MR}}_{i}\right)\right)$$

#### Data preparation and pre-process

Several datasets were used to estimate global cropland WUE, including satellite data and reanalysis meteorological data for GPP and NPP estimation, ET dataset, cropland distribution data, flux tower observations and ancillary data (Table 3).

**Table 3:** Main input datasets used to estimate global cropland water-use efficiency in this study.

Dataset	Variable	Spatial Resolution	Temporal Resolution
ERA5	Air temperature	0.25° × 0.25°	1 h
	Surface solar radiation downward		
	Dew point temperature		
GLASS	*f*APAR	500 m	8 days
	LAI		
ETMonitor	Evapotranspiration (ET)	1 km	Daily
	Net radiation flux (Rn)		
	Soil heat flux (G)		
ESA CCI	Cropland Class	300 m	1 year

### Meteorological forcing dataset

The meteorological forcing data, including the downward shortwave radiation, air temperature, and dew-point temperature, were obtained from the ERA5 (fifth-generation ECMWF atmospheric reanalysis) dataset, (https://www.ecmwf.int/en/forecasts/datasets/reanalysis-datasets/era5)^39^. The ERA5 dataset with a temporal and spatial resolution of 1-h/0.25° was averaged to daily temporal resolution and downscaled to 1-km spatial resolution using statistical downscaling approaches^40^ based on the topographic information retrieved from the SRTM30 DEM^41^.

### Vegetation remote sensing dataset

The fraction of absorbed photosynthetically active radiation (*f*APAR)^42,43^ and leaf area index (LAI) data^44^ was obtained from the Global LAnd Surface Satellite (GLASS) products (https://glass.hku.hk/download.html). The spatial resolution of the GLASS *f*APAR and LAI data was 500 m, and they were upscaled to 1-km resolution using the average resampling method. The temporal resolution of GLASS *f*APAR and LAI was 8-day, and they were interpolated to daily steps by linear interpolation method for further estimation of daily GPP and crop maintenance respiration.

### ETMonitor dataset

Daily ET data were obtained from the ETMonitor product with daily and 1-km resolution (https://data.casearth.cn/dataset/6253cddc819aec49731a4bc2)^29,45,46^. Meanwhile, the daily soil heat flux (*G*) and net radiation flux (Rn) data were retrieved using machine learning methods during the application of ETMonitor for global ET estimation and they were used to estimate EF (characterizing the water availability constraint in the EF-LUE model) during GPP estimation in this study. The ETMonitor model is based on the mechanisms of energy balance, water balance, and plant physiological processes that govern the surface energy and water exchange processes^29,46,47^. The estimated daily ET was validated based on the global in situ observations at site scale across various ecosystems, with overall high correlation (0.75), low bias (0.08 mm d^−1^), and low root mean square error (0.93 mm d^−1^). Specifically for croplands, the correlation of daily ET from ETMonitor exceeds 0.7. The ETMonitor dataset capture the expected global ET patterns both in space and in time. Particularly, the ET dataset has better performance in capturing the spatial variation of ET in the irrigated cropland regions and mountain regions with complex terrain than other global ET products, e.g., the GLEAM and MOD16 ET products, which benefits the estimation of the cropland WUE.

### Cropland distribution data

Global cropland distribution was obtained from global annual land cover maps at 300-m spatial resolution from the European Space Agency Climate Change Initiative Land Cover (ESA CCI-LC) (http://maps.elie.ucl.ac.be/CCI/viewer/download.php)^48^. The global annual land cover data at 300-m spatial resolution were aggregated to 1-km spatial resolution using the majority principle. A 1-km pixel was classified as cropland if the cropland coverage of the 1-km pixel calculated from the 300-m resolution map was greater than 50%.

### Flux tower observations

**Fig. 3:**
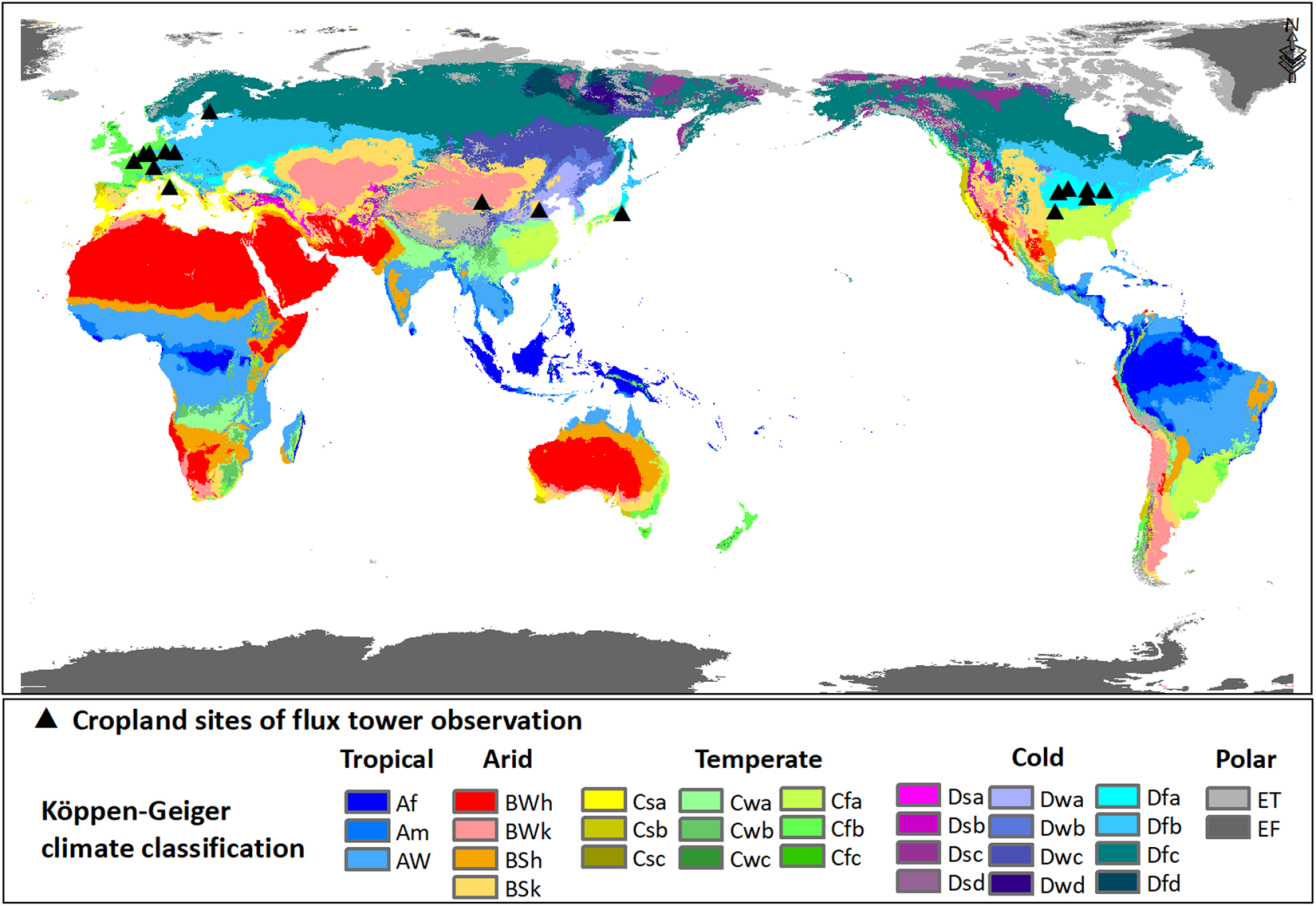
The cropland sites of flux tower observation on a map of Köppen climate zones used in the current study. Codes of climate zones in the color legend are adopted from Köppen-Geiger climate classification. Triangle symbols denote the cropland flux tower sites, ground data from which were used in this study.

The land surface flux tower observations at 21 cropland sites around the world were collected to calibrate and validate the estimated GPP and NPP and to validate the WUE estimation results. Details of the cropland flux tower sites in this study are given in Table 4 and site locations are illustrated in Fig. 3. These sites were from the global FLUXNET2015 dataset^49^ (https://fluxnet.org/data/fluxnet2015-dataset) and regional flux observation networks, e.g., AmeriFlux^50^ (https://ameriflux.lbl.gov/), AsiaFlux^51^ (https://db.cger.nies.go.jp/asiafluxdb/?page_id=16), ChinaFlux^52^ (http://www.chinaflux.org/).

**Table 4:** Cropland flux tower sites used in this study.

Site	Latitude	Longitude	MAT (°C)	MAP (mm)	Climate Zone	Periods	Crops
FI-Jok	60.90	23.51	4.6	627	Dfc	2001–2003	barley
CH-Oe2	47.29	7.73	9.8	1155	Dfb	2004–2014	winter wheat/winter barley/oilseed rape
DE-Geb	51.10	10.91	8.5	470	Dfb	2001–2014	barley/ oilseed rape/potatoes/summer maize
DE-Kli	50.89	13.52	7.6	842	Dfb	2005–2014	winter wheat/winter barley/oilseed rape
US-Ne1	41.17	−96.48	10.07	790.37	Dfa	2001–2012	maize
US-Ne2	41.16	−96.47	10.08	788.89	Dfa	2001–2012	maize/soybean
US-Ne3	41.18	−96.44	10.11	783.68	Dfa	2001–2012	maize/soybean
US-Br3	41.97	−93.69	8.9	846.6	Dfa	2006–2011	corn/soybean
US-Br1	41.97	−93.69	8.95	842.33	Dfa	2009–2011	corn/soybean
US-Bo1	40.01	−88.29	11.02	991.29	Dfa	2004–2005	maize/soybean
US-IB1	41.86	−88.22	9.18	929.23	Dfa	2009–2011	corn/soybean
US-CRT	41.63	−83.35	10.1	849	Dfa	2011–2013	soybean/winter wheat
IT-CA2	42.38	12.03	14	766	Csa	2011–2014	winter wheat
FR-Gri	48.84	1.95	12	650	Cfb	2004–2014	winter wheat/winter barley/summer maize
BE-Lon	50.55	4.75	10	800	Cfb	2004–2013	sugar beet/winter wheat/ potato
DE-RuS	50.87	6.45	10	700	Cfb	2012–2014	winter wheat/potatoes
DE-Seh	50.87	6.45	9.9	693	Cfb	2008–2010	winter wheat
US-ARM	36.61	−97.49	14.76	846	Cfa	2003–2012	rice
MSE	36.05	140.03	13.7	1200	Cfa	2004–2006	rice
DaMan	38.86	100.37	7.3	130.4	BWk	2015–2018	maize
YC	36.83	116.57	13.1	582	BSk	2004–2010	winter wheat/Summer maize

Climate zone codes were adopted from Köppen-Geiger climate classification. MAT: mean annual temperature; MAP: mean annual precipitation.

#### Auxiliary data

The climate classification map was used as auxiliary data for optimizing the EF-LUE model parameters globally. The climate zones were defined by the sub-types of the Köppen-Geiger climate classification (KGC)^35^, which includes a total of 5 major climate zones and 30 sub-types as shown in Fig. 3.

## Data Records

The Global 1-km cropland WUE dataset (GCWUE) is publicly available on the CASEarth Data Sharing and Service Portal (https://data.casearth.cn/dataset/640f0132819aec3f2b52a4bb?locale=en)^53^. This dataset contains global annual cropland WUE with a spatial grid resolution of 1 km, and the unit is g C/kg H_2_O/yr, from 2001 to 2020. This dataset uses the WGS84 coordinate system with latitude and longitude projection (EPSG: 4326). The data are stored in GeoTIFF format. Each file contains one data layer (band) of annual cropland WUE over croplands of the globe.

## Technical Validation

### Validation of the estimated GPP, NPP and WUE

The flux observations from the 21 global cropland ecosystem flux sites worldwide were used to obtain the site values of evapotranspiration and GPP, and the site NPP was obtained by combining the site GPP and the GLASS LAI data of the pixel of the corresponding sites (used to estimate crop maintenance respiration). Finally, the site values of cropland WUE were calculated and used as the “ground truth” data to validate the cropland WUE product. Further, we calculated the WUE based on GLASS (GLASS WUE) and MODIS (MODIS WUE) datasets and compared them with the WUE dataset developed in this study, since both GLASS and MODIS datasets provide NPP and ET variables.

**Fig. 4:**
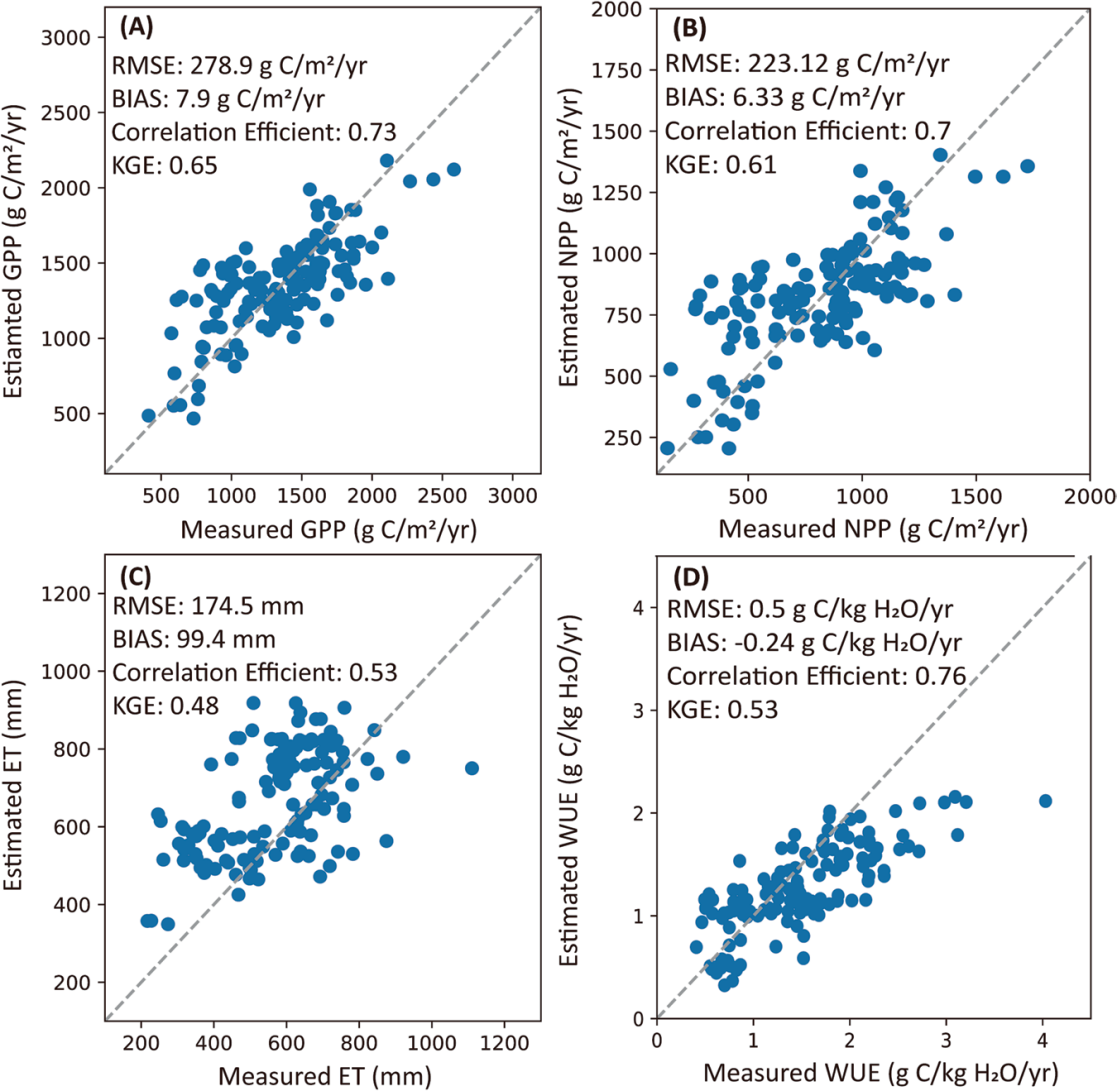
Comparisons of annual cropland GPP, NPP, ET and WUE between the estimates from the model in this study and ground measurements using eddy covariance system at cropland flux tower sites: (**A**) GPP; (**B**) NPP; (**C**) ET; (**D**) WUE.

The validation results show that the GPP, NPP, and WUE (referred to as GCWUE) all have a good performance that Pearson correlation efficiency (ρ) of annual cropland GPP was 0.73, the Root Mean Square Error (RMSE) was 278.9 g C/m^2^/yr, the BIAS was 7.92 g C/m^2^/yr, and the Kling-Gupta efficiency (KGE) was 0.65; the ρ of annual cropland NPP was 0.7, the RMSE was 223.12 g C/m^2^, the bias was 6.33 g C/m^2^/yr, and the KGE was 0.61; the ρ of annual cropland ET was 0.53, RMSE was 174.52 mm/yr, the BIAS was 99.4 mm/yr, and the KGE was 0.48; the ρ of annual cropland WUE was 0.76, the RMSE was 0.5 g C/kg H_2_O/yr, the bias was −0.24 g C/kg H_2_O/yr, and the KGE was 0.53 (Fig. 4, Table 5).

**Table 5:** Accuracy performance of GCWUE (developed in this study), MODIS WUE, and GLASS WUE evaluated using ground measurements by eddy covariance system at cropland flux tower sites.

Variable	Products	Correlation Efficient	Root Mean Square Error	BIAS
Net primary productivity	EF-LUE	0.70	223.12	6.33
	MODIS	−0.12	464.99	−302.83
	GLASS	0.16	415.04	−268.20
Evapotranspiration	ETMonitor	0.53	174.52	99.41
	MODIS	0.20	176.48	−64.08
	GLASS	0.50	202.07	142.31
Water use-efficiency	GCWUE	0.76	0.50	−0.24
	MODIS WUE	0.21	0.81	−0.48
	GLASS WUE	0.51	0.91	−0.70

The units for root mean square error and BIAS of net primary productivity, evapotranspiration and water use efficiency (WUE) are g C /m^2^ /yr, mm/yr, and g C/kg H_2_O/yr, respectively.

The GCWUE generated by this study outperforms the MODIS WUE and GLASS WUE (Table 5). The ρ of MODIS WUE and GLASS WUE were 0.21 and 0.16, respectively, both lower than GCWUE; the RMSE of MODIS WUE and GLASS WUE were 0.81 g C/kg H_2_O/yr and 0.91 g C/kg H_2_O/yr, respectively, both higher than GCWUE; the BIAS was −0.48 g C/kg H_2_O/yr and −0.70 g C /kg H_2_O/yr, both larger than GCWUE. For each climate zone, the GCWUE has better performances than GLASS WUE and MODIS WUE. Specifically, in the arid climate zone, the BIAS of GCWUE MODIS WUE and GLASS WUE are −0.18 g C /m^2^ /yr, −0.95 g C /m^2^ /yr, −1.06 g C /m^2^ /yr, respectively; in the temperate climate zone, they are −0.31 g C /m^2^ /yr, −0.45 g C /m^2^ /yr, −0.75 g C /m^2^ /yr, respectively; in the cold zone, they are −0.22 g C /m^2^ /yr, −0.44 g C /m^2^ /yr, −0.64 g C /m^2^ /yr, respectively.

We further validated the NPP and ET from MODIS and GLASS datasets using site flux observations (Table 5). The results show that MODIS severely underestimates the cropland NPP, and also underestimates ET to some extent, which is consistent with previous studies^54,55^. Severe underestimation of MODIS NPP leads to underestimation of its WUE. GLASS also underestimated the NPP of cropland, while GLASS overestimated ET, leading to the underestimation of WUE. The accuracy of the NPP generated by the optimization model for cropland in this study is significantly higher than that of MODIS and GLASS. The BIAS of MODIS NPP and GLASS NPP are both significantly larger than that of NPP generated in this study, which indicates systematic underestimations of NPP in MODIS and GLASS. And the performance of ET estimated by ETMonitor is also better than that of GLASS and MODIS, resulting in better estimated WUE in our study than MODIS WUE and GLASS WUE.

### Spatial and temporal variation of global cropland WUE of different products

**Fig. 5:**
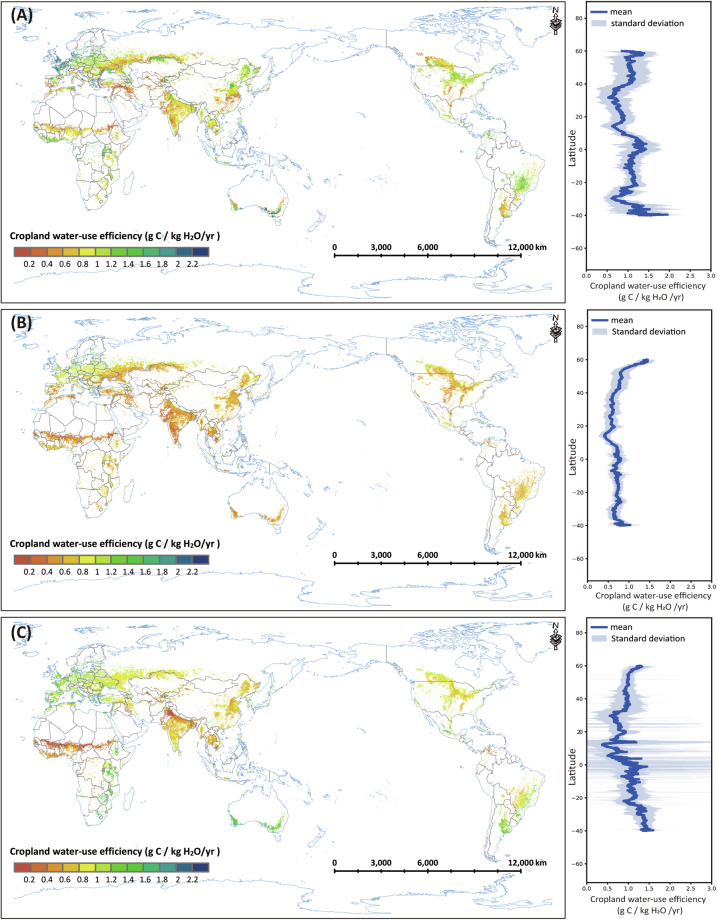
Spatial pattern (left panel) and latitudinal profile (right panel) of global mean annual cropland water-use efficiency (WUE) (averaged over 2001–2018): (**A**) GCWUE (generated by this study); (**B**) GLASS WUE; (3) MODIS WUE.

We calculated the mean annual GCWUE estimated by this study, MOD17, and GLASS products in the available common years (2001–2018), and compared the spatial pattern of the three datasets of cropland WUE (Fig. 5). In general, GCWUE, MOD17 WUE, and GLASS WUE show similar spatial patterns. The areas with high WUE are mainly located in the tropical region (e.g. Brazil, West Africa, Southeast Asia), northern Europe, and Austria; the areas with low WUE are mainly located in the Sahel region and India (about 10° N - 20° N).

**Fig. 6:**
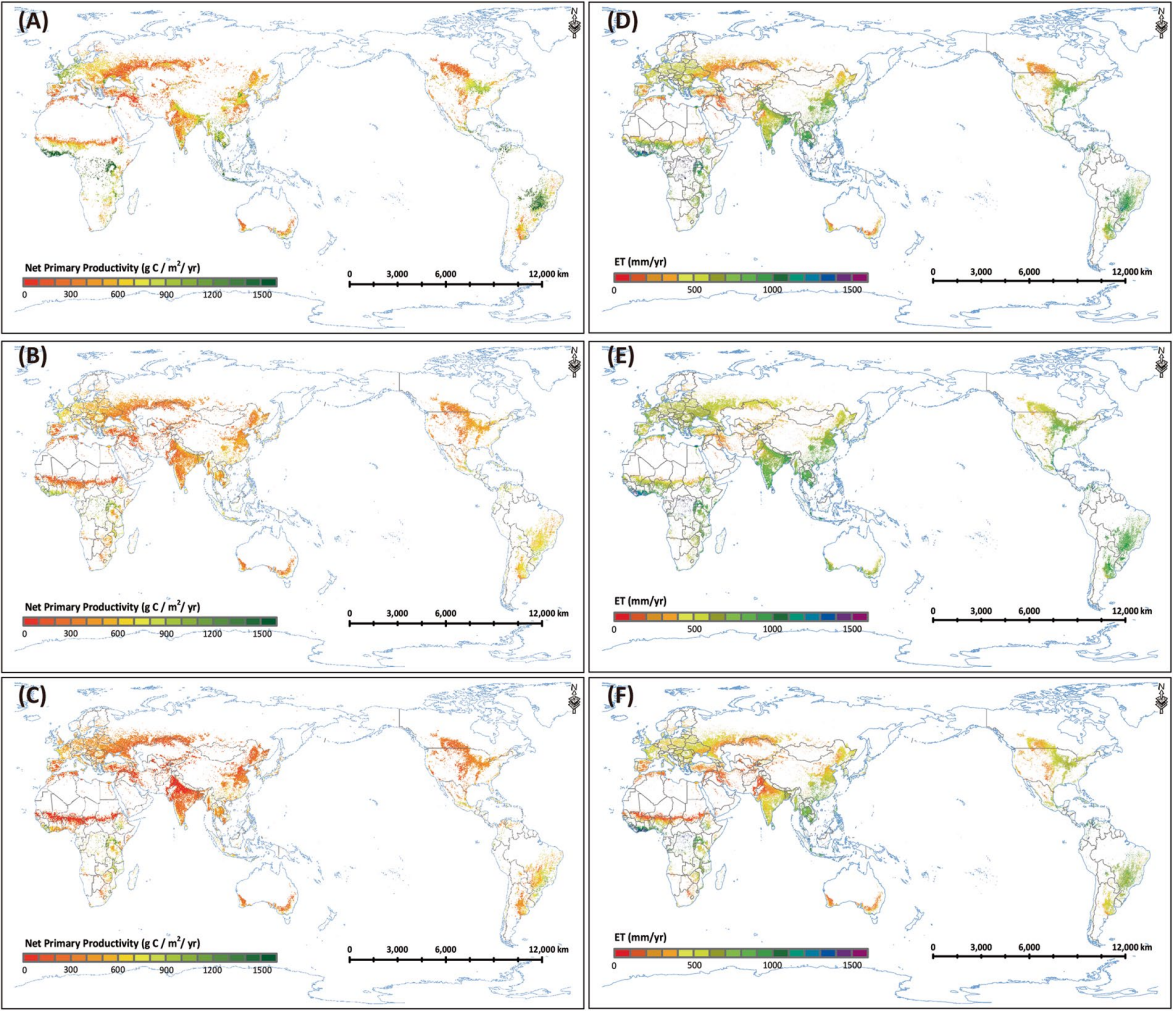
Spatial pattern of global mean annual cropland NPP and ET (averaged over 2001–2018): (**A**) EF-LUE NPP (generated by this study); (**B**) GLASS NPP; (**C**) MODIS NPP; (**D**) ETMonitor ET; (**E**) GLASS ET; (**F**) MODIS ET.

Similar to the site-level results, the MODIS WUE is generally lower than GCWUE, especially in India and the North China Plain with large irrigated cropland (Fig. 5), because the MODIS dataset has been shown to underestimate NPP (Fig. 6), especially for irrigated crops^54,55^. GLASS WUE is generally lower than GCWUE and MODIS WUE, especially in India, Russia, and Australia, because the GLASS NPP is underestimated, and the GLASS ET is overestimated (Fig. 6). One of the reasons for the underestimation of GPP and NPP of MODIS and GLASS is that their GPP estimation methods assumed a constant ε_max_ (1.044 g C MJ^−1^ in MOD17) for all crops in all climate zones. In this study, the improved EF-LUE GPP model, which was used to estimate GPP and then NPP, was calibrated using the available cropland flux tower observation data across different climate zones and the corresponding parameters for each climate zone were applied to global GPP estimation, overcoming the severe underestimation of GPP.

**Fig. 7:**
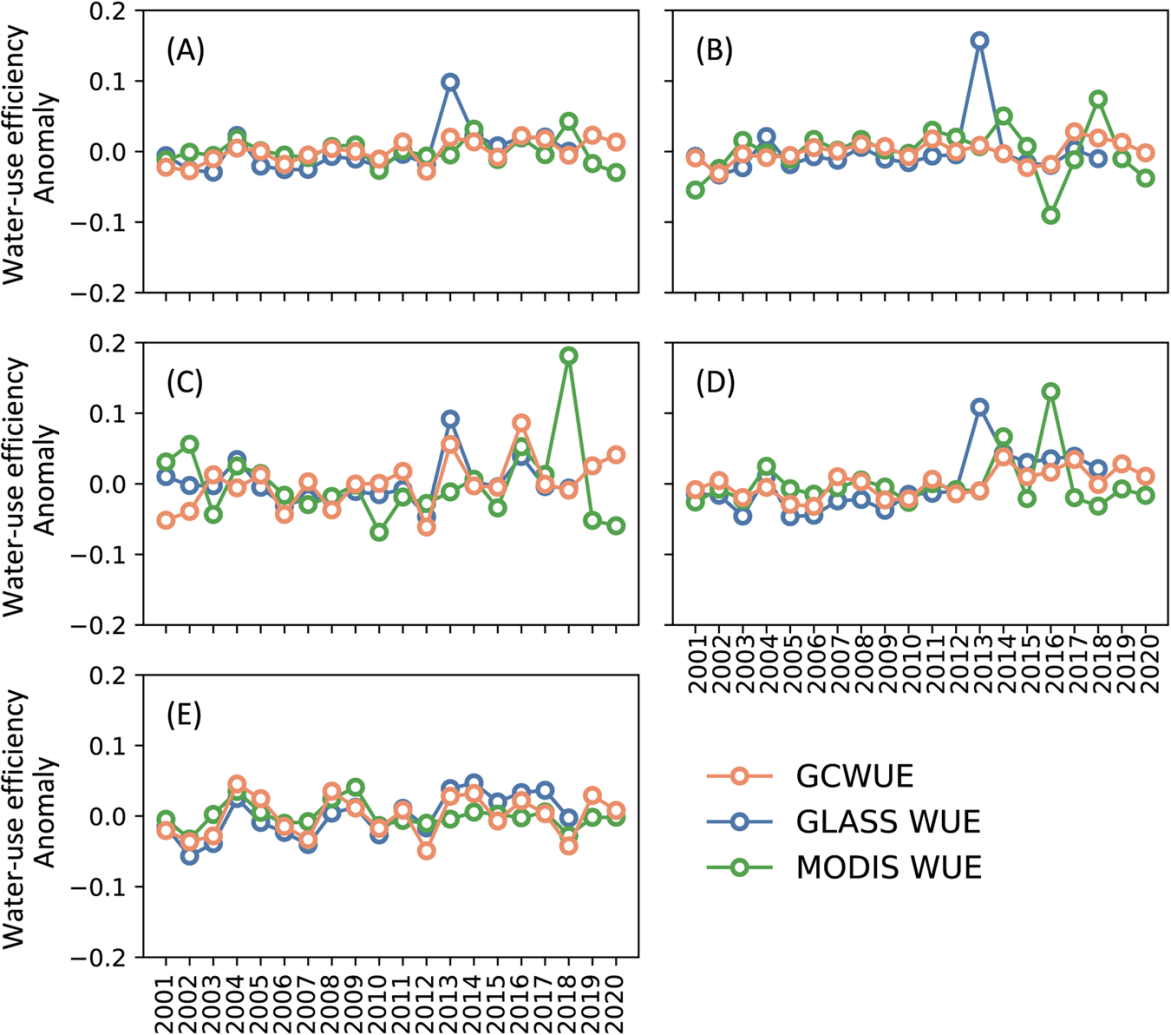
Anomaly of cropland water-use efficiency (WUE) by this study (GCWUE), GLASS (GLASS WUE), and MODIS (MODIS WUE) datasets in global and climatic zones (main 4 classes in Köppen-Geiger classifications): (**A**) Global; (**B**) Tropical; (**C**) Arid; (**D**) Temperate; (**E**) Cold.

In addition, we compared the anomaly changes in WUE of these three products (Fig. 7). The results show that the WUE temporal change pattern of the three products is similar at the global and different climate zone scales, which reflect the responses of cropland ecosystem to climate variations. For instance, WUE from three products all declined around 2015 in globe (Fig. 7A), in the tropical zone (Fig. 7B) and in the arid zone (Fig. 7C), likely due to the strong 2015 El Niño (October 2014 - April 2016). This event caused severe global droughts, reducing crop yields and thus lowering cropland WUE^56^. Moreover, the interannual variability of WUE varies across climate zones due to different sensitivity to climate variations. Arid zones, being highly sensitive, experience larger WUE fluctuations than other climate zones. These fluctuations suggest these cropland WUE products can reflect ecosystems’ response to climate variations to some extent, and the estimation products can capture the temporal changes in the cropland WUE. Compared with GLASS WUE and MODIS WUE, the GCWUE developed in this study has far fewer extreme fluctuation points possibly caused by data uncertainties (e.g., the values in 2013 in the global and tropical time series of GLASS WUE and in the arid zone time series of MODIS WUE). This indicates that the GCWUE product is more reliable and robust in time series. The main reason may be that GCWUE unified the ET and NPP estimation model input data sources and considered the carbon-water coupling process in the WUE estimation model, reducing abnormal values in WUE estimation.

## Usage Notes

In the present study, we developed a long-term global cropland WUE dataset with a spatial resolution of 1-km from 2001 to 2020. The validation showed its high accuracy (e.g., RMSE = 0.5 g C/kg H_2_O/yr) and ability to capture the spatial and temporal variation of global cropland WUE. However, the uncertainty associated with the algorithm and the forcing data should be noticed:

- The various uncertainties associated with the estimation of GPP, NPP, crop respiration, and ET due to the complexity of factors affecting the plants are propagated to the estimate of WUE. For example, the estimation of respiration in the current study is based on empirical Biome Properties Lookup Table (BPLUT) parameterizations using the growth and maintenance paradigm that relates respiration rate to temperature and the combination of mass, Nitrogen (N), photosynthetic capacity, photosynthetic Carbon (C) gain, and growth rate^57,58^. This respiration estimation paradigm assumes that the relative contributions of the different core biological functions of respiration are in equilibrium and that enzyme capacities do not change with time, which remains poorly studied^37,59^. Meanwhile, there are other environmental and biological factors that regulate respiration and carbon flux, e.g., the salinity, leaf carbohydrate rate, etc., that could not be captured in the current parameterizations.
- To reduce the uncertainty associated with GPP estimation, we optimized the model parameters of the EF-LUE model using available data from cropland flux tower sites in different climate zones and applied the corresponding model parameters for each climate zone to conduct global GPP estimation. However, the number of cropland flux tower sites is limited, and their spatial distribution is uneven. Most sites are in the boreal and temperate regions of the Northern Hemisphere, with few sites in the Southern Hemisphere, especially in Africa and South America. The limited number and uneven distribution of flux tower sites inevitably lead to uncertainties in large-scale GPP estimation, which in turn lead to uncertainties in global WUE estimation.
- The mismatch between the footprint of the tower-based flux observations and the pixel size of the remote sensing data will also contribute to the uncertainty during the GPP model calibration and validation procedures, which in turn will affect the accuracy of the WUE product generated in this study. The flux tower is several hundred meters depending on the wind direction, atmospheric stability, and land surface type, while the pixel size in this study is 1-km.
- The accuracy of the WUE is also limited by the lack of detailed information on crop types or rotations. This may be more severe in regions with complex cropping systems and multiple growing seasons. Our model optimization somehow allows us to partially identify the differences in crop characteristics or parameters in different climate zones during GPP estimation, which is helpful to improve the accuracy of WUE estimate. However, it should be noted that in the regions with multiple growing seasons, different crops may be planted in different seasons (e.g., rotation of maize and wheat in the North China Plain), and their properties may differ significantly (e.g., C3 and C4 pathways), which is difficult to be captured in this study because reliable crop type map or rotation information is not available.
- The forcing data also partly explain the uncertainty in WUE. For example, a previous study showed that different *f*APAR datasets showed large inconsistencies in spatial patterns in both multi-year averages and interannual trends, which could lead to large differences in estimated GPP^60^. In particular, different *f*APAR datasets were locally optimal over some regions, and there is no general agreement on which *f*APAR product is best for global cropland application. Uncertainties in the *f*APAR data generate uncertainties in the estimation of GPP and NPP, which in turn propagates to the estimated cropland WUE in this study. Due to the inherent uncertainties in large-scale model forcing data, some extreme WUE estimates may also occur. According to the WUE calculated from site data and referencing previous studies, we set WUE (NPP/ET) values exceeding 50 g C/kg H_2_O/yr as outliers and set them as “no data” in the dataset.
- In addition, we used the distribution of cropland derived from the ESA CCI LULC dataset as a mask for cropland GPP estimation, and the misclassification of cropland in this LULC dataset can also be transferred to WUE. In addition, LULC distribution is also an important input data for ET estimation, and the MODIS land cover dataset has been adopted by ETMonitor for the global ET estimation^29^. The difference in cropland distribution between MODIS land cover and ESA CCI land cover can lead to a mismatch between estimated ET and estimated GPP at the pixel level, which introduces uncertainties in the estimation of cropland WUE.

## Data Availability

The codes used in this study are available at https://github.com/MinChiangRunner/GlobalCroplandWUE.git.
